# Next‐generation sequencing in precision oncology: Patient understanding and expectations

**DOI:** 10.1002/cam4.1947

**Published:** 2019-01-01

**Authors:** J. Scott Roberts, Michele C. Gornick, Lan Q. Le, Natalie J. Bartnik, Brian J. Zikmund-Fisher, Arul M. Chinnaiyan, for the MI‐ONCOSEQ Study team, Dan Robinson, Yi‐Mi Wu, Chandan Kumar, Xuhong Cao, Jyoti Athanikar, Erica Rabban, Janice Griggs, Robert Lonigro, Marcin Cieslik, Pankaj Vats, Priya Kunju, Javed Siddiqui, Mandy Howery, Yu Ning, Rui Wang, Fengyun Su

**Affiliations:** ^1^ Department of Health Behavior and Health Education University of Michigan School of Public Health Ann Arbor Michigan; ^2^ Center for Bioethics & Social Sciences in Medicine University of Michigan Medical School Ann Arbor Michigan; ^3^ Rogel Cancer Center University of Michigan Medical School Ann Arbor Michigan; ^4^ Department of Internal Medicine University of Michigan Medical School Ann Arbor Michigan; ^5^ Department of Pathology University of Michigan Medical School Ann Arbor Michigan; ^6^ Michigan Center for Translational Pathology University of Michigan Medical School Ann Arbor Michigan; ^7^ Department of Urology University of Michigan Medical School Ann Arbor Michigan; ^8^ Howard Hughes Medical Institute University of Michigan Medical School Ann Arbor Michigan

**Keywords:** genome sequencing, informed consent, patient education, precision oncology

## Abstract

**Background:**

Implementation of precision oncology interventions poses several challenges to informed consent and patient education. This study assessed cancer patients’ understanding, expectations, and outcomes regarding participation in research examining the impact of matched tumor and germline sequencing on their clinical care.

**Methods:**

A total of 297 patients (mean age: 59 years; 50% female; 96% white) with refractory, metastatic cancer were surveyed, including 217 who completed surveys both before and after undergoing integrated whole exome and transcriptome sequencing as part of a larger clinical research study.

**Results:**

At baseline, the vast majority of patients expected to receive several potential direct benefits from study participation, including written reports of sequencing findings (88%), greater understanding of the causes of their cancer (74%), and participation in clinical trials for which sequencing results would make them eligible (84%). In most cases, these benefits were not realized by study completion. Despite explanations from study personnel to the contrary, most participants (67%‐76%) presumed that incidental germline sequencing findings relevant to noncancerous health conditions (eg, diabetes) would automatically be disclosed to them. Patients reported low levels of concern about study risks at baseline and low levels of regret about study participation at follow‐up.

**Conclusions:**

Findings suggest that cancer patients participating in precision oncology intervention research have largely unfulfilled expectations of direct benefits related to their study participation. Increased focus on patient education to supplement the informed consent process may help manage patients’ expectations regarding the extent and likelihood of benefits received as a result of undergoing genomic sequencing.

## 1 BACKGROUND

Next‐generation sequencing is rapidly emerging as a promising approach in cancer treatment. Improved understanding of somatic and germline aberrations involved in cancer pathology can inform selection of therapies tailored to the individual patient's underlying molecular lesions.^1^ Such interventions have already demonstrated utility in a small subset of cases of both adult and pediatric cancer,^2^, ^3^ and significant public and private investments are being made in a precision oncology model of care for future cancer patients.^4^

For the promise of precision oncology to be realized, patients will need to understand its benefits, risks, and limitations prior to consenting to treatment. Yet integration of matched tumor and germline sequencing into clinical care presenting significant challenges for patient education.^5^ For example, limitations in genetic literacy can complicate provider‐patient communication of the purpose and findings of biomarker‐based therapies.^6^ Germline analyses can yield secondary or incidental results with health implications for both patients and family members, creating challenges for informed consent.^7^ Many current sequencing efforts are being used to identify clinical trials for which patients may be eligible, potentially blurring the line between research and clinical practice and giving rise to “therapeutic misconceptions” among patients.^8^ These challenges suggest a need for research to illuminate cancer patients’ appraisal and understanding of next‐generation sequencing, thereby informing future patient education and counseling efforts in this area.

Only a few investigations have explored patient perspectives in the context of precision oncology. For example, studies have examined decisions whether or not to participate in cancer genome sequencing studies^9^ and perceived benefits of genomic tumor profiling.^10^ However, most studies in this area have been cross‐sectional in nature and/or with relatively small sample sizes. Furthermore, it is unclear whether and to what extent patients lack awareness of—or hold misconceptions about—the use of genome sequencing to tailor treatment recommendations. To help address this gap, we report here on a longitudinal survey of patients undergoing integrated tumor and germline sequencing to inform treatment of their advanced or refractory cancer.

## 2 METHODS

### 2.1 Overview

This paper reports on results from an ancillary study embedded with the larger MI‐ONCOSEQ (Michigan Oncology Sequencing) program.^11^, ^12^ Briefly, MI‐ONCOSEQ is a precision oncology research protocol at the University of Michigan Comprehensive Cancer Center. It expansively profiles genetic aberrations of a patient's tumor through comparison to a matched normal sample. MI‐ONCOSEQ's approach allows for the identification of somatic and germline events, point mutations, amplifications, insertions/deletions, gene fusions, and outlier gene expression. Team members present sequencing results at an institutional precision medicine tumor board (PMTB) composed of members with expertise in medical oncology, hematology, clinical pathology, cancer genetics, genetic counseling, bioinformatics, and bioethics. The PMTB discusses potentially actionable findings, with a summary test report ultimately e‐mailed to each referring oncologist within approximately 1 week of the PMTB meeting. Actionable findings typically involved tumor sequencing (ie, somatic) results that might inform care or confer eligibility for cancer treatment trials, but also included germline results that could inform cancer prevention efforts in participants’ blood relatives.^13^ The referring oncologist then decides whether, how, and when to return sequencing results to patients and integrate these findings into the treatment plan.

### 2.2 Informed consent process for sequencing

A study team member met in person with each MI‐ONCOSEQ participant to review the parent project's purpose, procedures, and potential risks/benefits. The study's main goal was described as “to identify key genes important to cancer cells that could potentially influence clinical decision making for managing cancer.” Participants were informed that they would always be told about sequencing results that “have a direct impact on care of your current cancer,” but that “the results are not guaranteed to help your doctor take care of you.” As part of the consent process, participants chose whether or not they would want to receive secondary sequencing findings unrelated to the treatment of their current cancer, but that might inform future treatment of other cancers, either for themselves or blood relatives (eg, *BRCA *1/2 mutations). Study participation length was considered indefinite, given that participants’ tumor tissue would be stored for potential future studies and might be used to create cell lines for ongoing research that “may improve the lives of future patients with cancer.”

### 2.3 Participants and survey procedures

**Figure 1:**
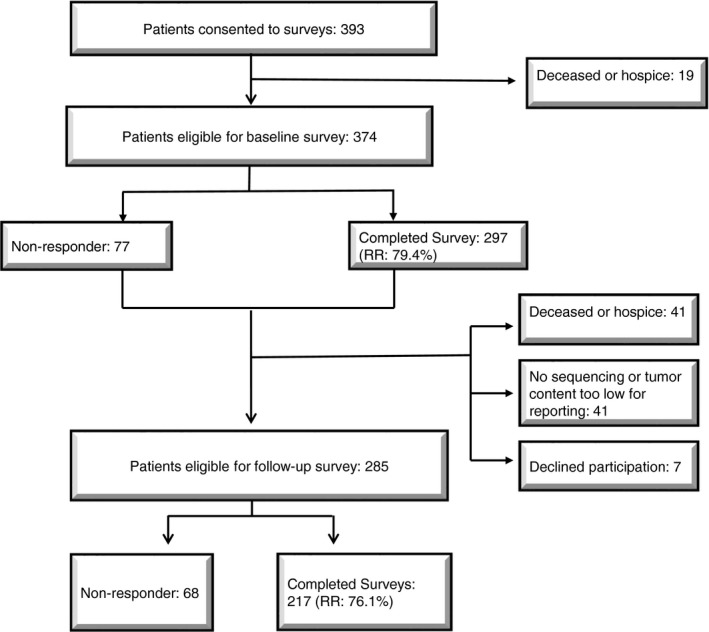
Study flowchart. RR, response rate

We surveyed adult patients (n = 297) with advanced stage solid tumor malignancies who enrolled in the MI‐ONCOSEQ program from April 2014 to December 2016. All patients participating in MI‐ONCOSEQ during that timeframe were eligible for our survey study, and the survey study sample did not differ from the broader MI‐ONCOSEQ patient population in terms of key demographic variables. The University of Michigan Medical School's Institutional Review Board (IRBMED) approved all study procedures. Surveys were administered at two time points: baseline and follow‐up. Study team members distributed baseline surveys in‐person to patients (response rate = 79%), after a study team member had obtained informed consent for the project. Patients were asked to complete and return the baseline survey within 2 weeks by mail. A web survey option was made available for patients preferring that format. The study team mailed follow‐up surveys home to patients approximately 2 weeks after the referring oncologist received their sequencing results surveys (n = 217; response rate = 76%). Some patients did not receive follow‐up surveys because their tumor biopsy material could not be properly analyzed (most follow‐up survey items presumed that the patient's sequencing report had been generated), or because they died in the period between the baseline and follow‐up surveys (see Figure 1 for more description of the flow of study procedures and attrition over the course of the longitudinal survey).

### 2.4 Measures

The patient surveys used both validated self‐report measures and questionnaire items created specifically for this study. We developed the latter with the assistance of a multidisciplinary team of oncologists, survey methodologists, genetic counselors, bioethicists, and health communication experts. Surveys were designed to minimize participant burden (ie, taking <30 minutes to complete).

#### 2.4.1 Patient characteristics

Demographic characteristics (eg, age, gender, and level of education) were assessed via standard self‐report questionnaire items. We gathered information about the patient's cancer (type, stage) from the PMTB test report. A single survey item asked participants to rate their current level of distress on a 0‐10 scale (10 being highest).

#### 2.4.2 Motivations and concerns regarding study participation

We assessed *motivations for study participation* by posing nine reasons why cancer patients might participate in genome sequencing and asking participants to indicate how much they agreed or disagreed (1 = strongly disagree to 4 = strongly agree) with each statement. Items encompassed reasons including potential direct clinical benefits, altruism, and social network pressure. Participants also identified which item reflected their main reason for joining the study. We generated an overall index of motivations for study participation by summing items (possible range: 9‐36, with higher scores indicating higher levels of motivation), with a subscale consisting of items related to direct clinical benefits. This scale demonstrated good reliability in the current study, with a Cronbach alpha score of 0.81.

*Concerns about study participation* were assessed via six items asking participants to rate their level of concern (1 = not at all to 5 = extremely) about potential negative aspects of study participation. Items addressed issues including lack of direct benefit from sequencing results and distress from learning genomic information.

#### 2.4.3 Knowledge

Baseline study surveys assessed patients’ objective and subjective knowledge of genome sequencing, including how it would be employed in the MI‐ONCOSEQ project.

##### Knowledge of genome sequencing (objective)

We adapted a general knowledge of genome sequencing scale^14^ for use in a cancer population. Six true/false items assessed knowledge of basic facts about genome sequencing, including its potential benefits and limitations. We created an overall knowledge score by summing correct responses to scale items (possible range: 0‐6, with higher scores indicating greater knowledge).

##### Knowledge of informed consent (subjective)

We adapted an existing measure assessing the quality of informed consent in clinical cancer research^15^ for use in this study. Six items asked participants to rate how well they understood (1 = “I did not understand this at all” to 5 = “I understood this very well”) different aspects of the MI‐ONCOSEQ research project, including study purpose, procedures, and risks and benefits. Items were summed to create an overall measure of subjective understanding of informed consent (possible range: 6‐30 with higher scores indicating higher level of understanding). The scale demonstrated good reliability in the current study, with a Cronbach alpha score of 0.88.

##### Knowledge of study's return of results policies (objective)

We assessed participants’ knowledge of genomic results to be returned to them in the MI‐ONCOSEQ project using a novel 10‐item scale. Participants received a list of possible types of genome sequencing results and indicated whether or not they would receive such results as part of their study participation. Response options included “I would receive these results automatically,” “I would only receive these results if I said I wanted them”, “I would not receive these results,” and “Not sure.” We created an overall knowledge score by summing correct responses to scale items (possible range: 0‐10, with higher scores indicating greater knowledge).

#### 2.4.4 Expectations of study benefits

An 8‐item measure, administered at baseline, assessed patients’ expectations regarding potential study benefits. Questions asked participants about the extent to which they agreed (1 = strongly disagree to 4 = strongly agree) with various statements expressing expectations for study participation. Items addressed what type of information they expected to gain from sequencing, how they expected the results to be communicated, and potential outcomes such as clinical trial enrollment. The follow‐up survey posed the same eight items, and participants were asked whether or not the outcome described in the survey item had occurred (response options included: “yes,” “no,” or “unsure”). For the baseline survey, we summed items to create an overall measure of expectations of study benefits (possible range: 8‐32, with higher scores indicating higher levels of expectation). The scale demonstrated good reliability in the current study, with a Cronbach alpha score of 0.83.

#### 2.4.5 Decisional satisfaction

In the follow‐up survey, we administered two items from a validated measure of decisional satisfaction^16^ to assess participants’ satisfaction with (vs regret about) their decision to participate in the MI‐ONCOSEQ project. Items asked whether participants would undergo genome sequencing if they had the choice to do it again (1 = strongly disagree to 5 = strongly agree), and the extent to which they were satisfied with their decision to participate in the study (1 = not at all to 5 = extremely).

### 2.5 Data analysis

Descriptive statistics were used to (a) characterize the study sample in terms of its demographics and other characteristics and (b) report on item‐level responses across study measures including motivations and concerns regarding study participants, knowledge of return of results policies, and expectations regarding study benefits. Within‐subject analyses were performed for comparison of baseline and follow‐up survey responses on the benefit expectations measure, using only data from the 205 respondents who had completed these survey scales at both time points.

In post hoc analyses, we performed stepwise binary logistic regression using the forward logistic regression method (forward LR) for including variables in the model to assess predictors of key study outcomes including motivations for study participation, expectations of direct study benefits, and knowledge of study policies regarding return of results, with statistical significance assessed at *P* < 0.05. Each model contained seven predictors as follows: genomic sequencing knowledge, informed consent understanding, and psychological distress (measured at baseline), as well as age, gender (female or male), self‐reported race (white or non‐white), education (three categories: high school or less, some college, or college or greater). We excluded from analysis participants with missing values on the outcome measures or key covariates (<5%). Given the non‐normal distribution of scores on study outcome measures, we used a median split procedure to dichotomize scale scores for the following variables: motivations for study participation, expectations of direct study benefits, and knowledge of study policies regarding return of results. Logistic regression analyses yielded odds ratios reporting the odds of scoring in the higher vs lower group on motivations, expectations, and knowledge outcome measures.

## 3 RESULTS

### 3.1 Sample characteristics

**Figure 2:**
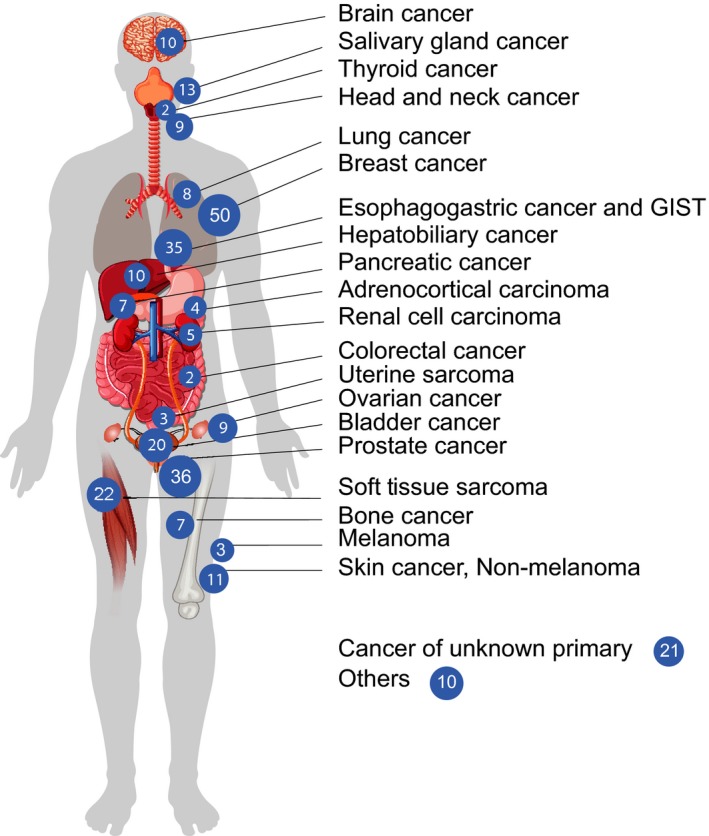
Cancer types and frequencies among study participants

Characteristics of the study sample are presented in Table 1. In brief, the sample was predominantly white (93.6%), middle aged (mean age = 57.9 years), and well‐educated (81.5% with at least some college education), with a roughly equal number of men and women (51% male vs 49% female). Patients’ cancer diagnoses included >20 types of cancer, with breast (n = 50) and prostate (n = 36) cancer as the most common types (see Figure 2 for more details).

**Table 1:** Sample characteristics (N = 297)

Characteristics	n (%)
Mean age, y (SD)	59.1 (12.0)
Range	20‐85
Gender	
Male	148 (50.2%)
Female	147 (49.8%)
Race/ethnicity	
White	285 (96.0%)
Black	3 (1.0%)
Other	5 (1.7%)
Highest level of education	
High school or less	55 (18.5%)
Some college	103 (34.7%)
College graduate or greater	139 (46.8%)
Mean psychological distress (0‐10 scale), SD	4.1 (2.7)

### 3.2 Motivations and concerns regarding study participation

Participants endorsed a range of motivations for study participation (see Table 2), including desire to contribute to cancer research, to assist future cancer patients, and to potentially obtain direct study benefits pertaining to their own cancer treatment and identification of genetic cancer risks in blood relatives. When asked what their main reason was for study participation, about half of the participants endorsed items related to advancing the field of cancer research. For example, 29% endorsed “to help researchers better understand my type of cancer,” and 21% endorsed “to contribute to cancer research.” Nearly 40% of participants cited a main reason related to direct study benefits for themselves or family members. For example, 30% endorsed “to see if my sequence results could help make treatment decisions for me,” 6% endorsed “to gain information relevant for the health of my biological relatives,” and 3% endorsed “to have more certainty about my type of cancer.”

**Table 2:** Motivations and concerns regarding study participation (N = 297)

Motivations for joining study	Disagree (%)	Agree (%)	Strongly agree (%)	Most important^a^ (%)
To help researchers better understand how to treat my type of cancer	7 (2.4)	35 (11.8)	255 (85.9)	90 (30.3)
To see if my DNA sequencing results could help make cancer treatment decisions for me	10 (3.4)	63 (21.3)	223 (75.3)	86 (29.0)
To contribute to cancer research.	9 (3.0)	73 (24.6)	215 (72.4)	42 (14.1)
Because I feel like I am helping other cancer patients	12 (4.0)	82 (27.6)	203 (68.4)	17 (5.7)
To gain information that may be relevant to the health of my biological relatives	25 (8.5)	86 (29.0)	186 (62.6)	24 (8.1)
To have more certainty about my type of cancer	25 (8.5)	88 (29.7)	183 (61.8)	12 (4.0)
Because my doctor recommended the study	56 (18.9)	124 (41.9)	116 (39.2)	14 (4.7)
To learn about my genetic risk for diseases other than cancer	92 (27.3)	100 (33.7)	116 (39.1)	1 (0.3)
Because my family encouraged me to participate	122 (41.5)	111 (37.8)	61 (20.7)	0 (0)

Concerns about study participation (Scale: 1 = not at all to 5 = extremely concerned)	Mean (SD)
The DNA sequencing results may not guide my current cancer treatment care	2.75 (1.4)
The DNA sequencing results could give unwanted information about my biological relatives’ risk of cancer	2.19 (1.3)
The DNA sequencing results might be confusing or difficult to understand	2.23 (1.3)
The DNA sequencing results could give me information about my risk for other conditions that I may not want to know about	2.08 (1.2)
The DNA sequencing results might lead my doctor(s) to recommend things that I don't want to do	1.98 (1.2)
The DNA sequencing results might make me worried or anxious	1.91 (1.2)

a Four (1.3%) individuals selected more than one “most important” motivation; seven (2.3%) individuals did not endorse a “most important” motivation.

Most participants did not endorse strong concerns about the potential risks of study participation (see Table 2). Fewer than 10% of participants indicated they were extremely concerned about potential study risks including increased anxiety and receipt of unwanted genetic information. The most commonly endorsed concern was that sequencing results would not be informative for the patient's own cancer treatment, with 13% reporting they were “extremely concerned” about this possible study outcome.

In an adjusted multivariable model, patients with at least a college degree had lower odds than those with a high school education of reporting that direct benefits of study participation were a study motivation (OR = 0.44, 95% CI: 0.22‐0.96, *P* = 0.040). Higher self‐reported level of understanding of the informed consent process was also significantly associated with higher odds of endorsing direct benefits as motivation for study participation (OR = 1.09, 95% CI: 1.05‐1.18, *P* = 0.001).

### 3.3 Knowledge of genome sequencing and study informed consent process

Participants demonstrated generally good overall knowledge of basic facts about genome sequencing, with an average score of 5.3 (SD = 0.99) out of six items (88%) correct on a validated measure of genome sequencing knowledge. The vast majority of participants recognized that identification of cancer genes does not always lead to prevention or cure (96% correct), sequencing could give people information about disease risks beyond cancer (89% correct), and that sequencing results cannot provide exact estimates of cancer risk (85% correct). The survey item most often answered incorrectly was “sequencing all cancer genes is a routine test that doctors can order for most people with cancer,” with 17% of respondents erroneously endorsing as true.

Participants varied in the degree to which they reported understanding different aspects of the study's informed consent process. Higher levels of subjective understanding were reported regarding study risks and benefits, with 55%‐60% of participants indicating that they understood these study elements very well. Levels of subjective understanding were somewhat lower regarding study purpose and procedures. For example, participants reported the lowest levels of subjective understanding for what would happen to their biological samples after DNA sequencing was completed, with only 37.5% saying that they understood this very well. Overall, 44% said that they understood the study very well when they signed its consent form.

We identified significant gaps in understanding regarding the study's policies for return of genomic sequencing results (see Table 3). The mean score on this knowledge scale was 4.1 (SD = 1.5) items out of 10 correct. In general, participants evidenced a bias toward assuming that all types of genome sequencing results would automatically be returned. This meant that the vast majority of participants incorrectly indicated that they would receive incidental sequencing results related to genetic risk of noncancerous conditions, as well as pharmacogenomic findings to inform medication use in noncancerous conditions.

**Table 3:** Knowledge of study policies regarding return of individual research results (n = 297)

Survey items (type of result)	Correct response	N (% correct)
Results relevant for relatives’ risk of developing cancer	Would receive automatically or only if wanted	288 (97.7)
Results that show an increased risk for a different type of cancer	Would receive automatically or only if wanted	286 (96.6)
Results that could guide current cancer treatment	Would receive automatically	264 (89.2)
Results that provide information about response to cancer medication	Would receive automatically	234 (78.8)
Results that help to explain my cancer but do NOT guide treatment	Would receive automatically	231 (78.3)
Results that show an increased risk for noncancerous conditions that cannot be treated effectively (eg, Alzheimer's disease)	Would not receive	20 (6.8)
Results that show a virus (eg, HIV or HPV)	Would not receive	15 (5.1)
Results that provide information about response to noncancer medications	Would not receive	13 (4.4)
Results relevant for relatives’ risk of developing noncancerous conditions	Would not receive	12 (4.1)
Results that show an increased risk for noncancerous conditions that can be treated effectively (eg, diabetes and heart conditions)	Would not receive	11 (3.7)

Multivariate logistic regression indicated that patients who self‐reported higher levels of understanding of the informed consent process (vs those reporting lower levels of understanding) had higher odds of demonstrating correct knowledge about study policies regarding return of research results (OR = 1.09, 95% CI: 1.03‐1.15, *P* = 0.003). Table 4 presents full results for this and other regression models conducted as part of the study.

**Table 4:** Logistic regression analyses of participant characteristics associated with key study outcomes

Participant characteristics	Perceived expectations of study benefits				Study motivations (direct benefits subscale)				Knowledge of study RoR policies			
	OR	95% CI		*P*‐value	OR	95% CI		*P*‐value	OR	95% CI		*P*‐value
Age	0.99	0.97	1.03	0.758	1.02	0.99	1.04	0.109	1.00	0.98	1.03	0.815
Gender												
Female	Ref	Ref	Ref	‐	Ref	Ref	Ref	‐	Ref	Ref	Ref	‐
Male	1.32	0.77	2.26	0.309	1.09	0.65	1.82	0.741	1.4	0.81	2.47	0.220
Race												
White	Ref	Ref	Ref	Ref	Ref	Ref	Ref		Ref	Ref	Ref	
Non‐white	0.93	0.26	3.34	0.908	0.82	0.27	2.45	0.724	1.12	0.35	3.57	0.844
Education												
High school or less	Ref	Ref	Ref	‐	Ref	Ref	Ref	‐	Ref	Ref	Ref	‐
Some college	0.91	0.38	2.12	0.828	0.53	0.23	1.19	0.123	0.75	0.32	1.75	0.508
College graduate or greater	0.76	0.29	1.56	0.361	0.44	0.20	0.96	*0.040*	0.66	0.29	1.50	0.320
Genome sequencing knowledge	0.77	0.60	1.03	0.082	1.04	0.79	1.38	0.773	1.24	0.94	1.63	0.125
Informed consent understanding	1.14	1.07	1.21	*0.001*	1.09	1.05	1.18	*0.001*	1.09	1.03	1.15	*0.003*
Psychological distress (baseline)	0.94	0.85	1.03	0.201	0.99	0.91	1.09	0.547	0.98	0.89	1.08	0.678

Italicized figures refer to statistically significant results (P < .05). RoR, return of results.

### 3.4 Expectations of study benefits

The vast majority of participants endorsed survey items at baseline indicating that they expected a wide range of potential study benefits, including written reports of sequencing findings (88%), discussions with physicians about the implications of sequencing results (92%), notifications of clinical trials for which they might be eligible (84%), and learning more about the causes of their own cancer (74%), as well as gene changes that might have implications for their relatives’ cancer risk (93%). In an adjusted multivariable model, self‐reported higher level of understanding of the study's informed consent process was significantly associated with greater odds of endorsing direct benefits from study participation (OR = 1.14, 95% CI: 1.07‐1.21, *P* = 0.001).

**Figure 3:**
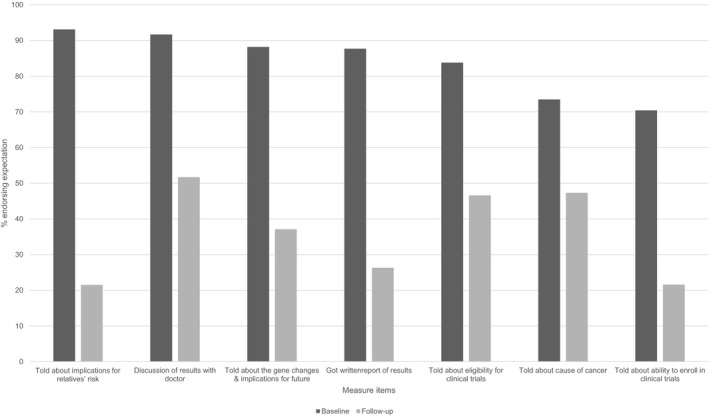
Expected vs realized study benefits. Survey items were considered endorsed if participants responded "agree" or "strongly agree" at baseline and "yes" at follow‐up"

In the follow‐up survey, many fewer participants indicated actually receiving such direct study benefits than had endorsed expecting them at baseline (see Figure 3). For example, only 22% said they had been told about gene changes related to their relatives’ cancer risk, 26% had gotten a written summary report of results, 47% had learned about clinical trials for which they might be eligible, and 52% had discussed sequencing results with their doctor. These analyses only included participants who had responded to relevant items in both the baseline and follow‐up surveys.

### 3.5 Decisional satisfaction and regret

At follow‐up, participants indicated high levels of satisfaction with their decision to take part in the genome sequencing study, with a mean score of 4.06 (SD = 1.1) on a 5‐point scale (1 = not at all to 5 = extremely satisfied). The vast majority of respondents also indicated that they would make the same choice to undergo sequencing if they had it to do over again (mean score = 4.37 (SD = 0.9) on 5‐point scale from 1 = strongly disagree to 5 = strongly agree).

## 4 DISCUSSION

This longitudinal survey is one of the first and largest studies to examine patients’ understanding and expectations regarding the use of next‐generation sequencing in their own cancer treatment. Perhaps our most notable finding was an apparent mismatch between patients’ pretest expectations of direct benefits from cancer genome sequencing and their actual realization of such benefits. Patients reported expecting a wide range of potential benefits from study participation, including greater understanding of underlying causes of their cancer, genomic information of relevance to their family members’ health, and the ability to enroll in clinical cancer trials on the basis of their sequencing results. Although in some cases these expectations were fulfilled, in more cases they were not. Patients’ high levels of satisfaction with their decision to participate in the study and low overall levels of decisional regret suggest that such unfulfilled expectations were likely not highly harmful. Yet one can imagine scenarios where unrealistically high patient expectations about the potential benefits of precision oncology studies (eg, to lead to life‐saving therapies for a terminal condition) could distort medical decision making or result in significant distress when such benefits do not occur (see Zikmund‐Fisher^17^ for a case example). Such potential “therapeutic misconceptions” about clinical research are not unique to precision oncology studies, but they may be encouraged by high levels of enthusiasm about the promise of genomically tailored approaches as expressed by the scientific community, for‐profit companies, and media coverage of the field.^18^ Knowledge and expectations were generally not associated with participants’ demographic characteristics (eg, age, gender, race), but they did correlate with how well participants felt they understood key aspects of the study.

Despite generally good knowledge about cancer genome sequencing in general, participants reported lack of awareness about key aspects of this genomic sequencing study. For example, fewer than 40% said they understood well what would happen to their biological samples after DNA sequencing was completed. Given common practices (and even federal requirements) of biobanking DNA samples for future, unspecified research, it may be prudent to provide study participants greater detail about how their samples might be used in future studies. Some states’ experiences with biobanking of residual blood spots from newborn screening show how public trust in the research enterprise can be damaged when individuals discover that their biological samples are being used for research purposes of which they were initially unaware.^19^

Another area prone to potential misunderstanding involves return of individual genomic research results. There has been considerable debate within the genomics research community about if, whether, and when study investigators have an obligation to disclose individuals’ genomic findings that are clinically or personally significant.^20^ In precision oncology research, practices have varied widely, with some studies not returning results at all, some disclosing only cancer‐specific findings, and others reporting on notable secondary findings (eg, germline findings suggesting high risk of inherited cardiovascular disorders).^21^ In this study, the policy was to return clinically significant findings from both germline and tumor sequencing that were cancer‐related, but not to return secondary findings for noncancerous conditions. Yet most patients appeared to presume they would be notified of *any* clinically significant secondary findings, even for diseases such as diabetes and Alzheimer's disease. Such beliefs may reflect the fact that patients don't always make sharp distinctions between clinical research and clinical practice (where the tendency would be to disclose clinically significant secondary findings), as well as an underappreciation of the complexity and labor involved to “hunt” for genomic findings unrelated to the initial purpose of sequencing.^22^ In addition, many studies do not explicitly inform participants about return of results policies for the multiple different types of secondary findings that might be generated.

Study findings suggest several areas in which patient education might be improved. One obvious point of intervention would be during informed consent processes where cancer genomic sequencing is taking place. It is worth pointing out that the misunderstandings observed in this study occurred despite a relatively well‐educated patient population and a consent process carried out by well‐trained study staff and genetic counselors. As many commentators have pointed out, informed consent processes for genomic research have often been overburdened.^23^, ^24^ It is not wise to expect that a severely ill population—one undergoing many life stresses and medical procedures—is going to fully absorb in one encounter the highly complex material represented by most precision oncology studies. As such, we would recommend use of a variety of communication strategies, some of which we have begun to implement at our own site. These include creation of a one‐page “Frequently Asked Questions” sheet as take‐home material to accompany the lengthy study consent form, and use of sample vignettes and visual aids to clarify the flow of study procedures and likelihood of direct benefits related to clinical care. Decision aids could also be useful here, and online aids specific to genome sequencing have now been developed.^25^

Although our study highlighted areas for improvement in patient education, it should be noted that participants in the study showed good knowledge of many basic facts about cancer genomic sequencing. For example, most recognized that identification of cancer genes does not inevitably lead to prevention or cure, that sequencing could generate secondary findings related to noncancerous conditions, and that sequencing results cannot provide highly precise estimates of cancer risk. Interestingly, the most commonly held misconception as assessed by our genome sequencing knowledge measure, as reported by nearly one in five respondents, was that cancer genome sequencing is a routine test within current clinical practice. Although literature on the ethical, legal, and social implications of genetic testing has often highlighted the potential harms of learning personal genetic information,^26^ relatively few patients in the study expressed concerns regarding the possibility of psychological distress or unwanted information as a result of undergoing sequencing. In fact, the most common concern of participants was that sequencing would not yield clinically useful findings, which seems well‐justified given that precision oncology studies have not yet demonstrated general effectiveness of molecular profiling in cancer treatment.^27^

Our findings should be interpreted in light of several study limitations. The study sample of cancer patients was disproportionately white and college‐educated, limiting generalizability of study results. Future research in this area should attempt to enroll patient populations with greater diversity in terms of race, ethnicity, and socioeconomic status. Another study limitation was that some key measures had either not been formally validated (eg, knowledge of study return of results policies) or were not administered in their entirety (eg, decisional regret), which may have introduced some measurement error into the assessment of key outcomes. In addition, some commentators have noted that surveys such as ours that demonstrate participant desires for direct study benefits are not inherently indicative of therapeutic misconception; they point out that patients can simultaneously hope that research participation will improve their own personal care while recognizing that such clinical benefits are not the primary purpose of a given study.^28^

In conclusion, this study highlights the need for careful communication with cancer patients considering the use of genome sequencing to inform their treatment plans. Clarifying the likelihood of clinical benefit from sequencing and the “what, when and how” of reporting sequencing results are essential to managing patient expectations and ensuring truly informed consent. As genome sequencing is increasingly incorporated in precision oncology, we must pay sufficient attention to the critical role that health communications can have in the patient experience.

## CONFLICT OF INTEREST

Arul Chinnaiyan serves on the Scientific Advisory Board of Tempus, Incorporated. The other authors report no conflicts of interest.
